# Extrapolating Metabolic Savings in Running: Implications for Performance Predictions

**DOI:** 10.3389/fphys.2019.00079

**Published:** 2019-02-11

**Authors:** Shalaya Kipp, Rodger Kram, Wouter Hoogkamer

**Affiliations:** ^1^Department of Integrative Physiology, University of Colorado, Boulder, CO, United States; ^2^School of Kinesiology, University of British Columbia, Vancouver, BC, Canada

**Keywords:** energetic cost, locomotion, marathon, oxygen uptake, running economy

## Abstract

Training, footwear, nutrition, and racing strategies (i.e., drafting) have all been shown to reduce the metabolic cost of distance running (i.e., improve running economy). However, how these improvements in running economy (RE) quantitatively translate into faster running performance is less established. Here, we quantify how metabolic savings translate into faster running performance, considering both the inherent rate of oxygen uptake-velocity relation and the additional cost of overcoming air resistance when running overground. We collate and compare five existing equations for oxygen uptake-velocity relations across wide velocity ranges. Because the oxygen uptake vs. velocity relation is non-linear, for velocities slower than ∼3 m/s, the predicted percent improvement in velocity is slightly greater than the percent improvement in RE. For velocities faster than ∼3 m/s, the predicted percent improvement in velocity is less than the percent improvements in RE. At 5.5 m/s, i.e., world-class marathon pace, the predicted percent improvement in velocity is ∼2/3rds of the percent improvement in RE. For example, at 2:04 marathon pace, a 3% improvement in RE translates to a 1.97% faster velocity or 2:01:36, almost exactly equal to the recently set world record.

## Introduction

The remarkable 2:00:25 exhibition marathon in Monza, Italy in 2017 and the current world record time of 2:01:39 set in Berlin in 2018 by Eliud Kipchoge raise an intriguing question: can we predict improvements in endurance running performance based on improvements in running economy (RE)? Together with lactate threshold and maximal oxygen uptake (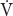
O_2_max), RE is one of the three primary physiological determinants of performance (Daniels, 1985; Joyner, 1991; Foster and Lucia, 2007). RE is traditionally defined as the rate of oxygen uptake (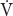
O_2_, in mlO_2_/kg/min) for running at a specified submaximal velocity^1^. Improvements in RE allow athletes to run at a faster velocity for the same oxygen uptake and thus achieve superior performances (Joyner, 1991; Hoogkamer et al., 2016, 2017). RE can also be expressed in oxygen uptake per unit distance (in mlO_2_/kg/km), by dividing 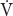
O_2_ by the running velocity at which it was assessed. From ∼2.2 to 5.6 m/s (8–20 km/h), net 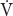
O_2_ (gross minus rest or standing) per distance remains fairly constant (Margaria et al., 1963; di Prampero et al., 1986; Saibene and Minetti, 2003; Lacour and Bourdin, 2015). Accordingly, 1% improvements in RE (lower rates) should directly translate to 1% faster running performances (Daniels and Daniels, 1992; McLaughlin et al., 2010). Indeed, we demonstrated that laboratory-measured percent changes in RE translate to similar percent changes in distance running performance (assessed by 3 km time trials) (Hoogkamer et al., 2016).

Recently, we have used these insights and models to translate metabolic savings reported in the literature (Hoogkamer et al., 2017) and measured in our laboratory (Hoogkamer et al., 2018a) into predicted improvements in elite marathon running performances. Unfortunately, the heterogeneity of racecourses, meteorological and competitive conditions, combined with fluctuations in the training status of elite marathon runners preclude controlled experiments on racing performance. Recent marathon race results suggest that finishing times may not match the theoretically predicted improvements from drafting (Hoogkamer et al., 2017) or advances in shoe technology (Hoogkamer et al., 2018a). Here, we examine the assumptions underlying our extrapolations and derive a revised model for extrapolating metabolic savings into running performance improvements.

## Running Velocity and $\dot{V}$

To extrapolate how changes in RE will impact performance, we focus on the gross 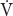
O_2_-velocity relation. If the relation is directly proportional with a zero 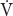
O_2_-intercept, running 1% faster exacts a 1% higher metabolic rate and we can expect that a 1% improvement in RE would allow for a 1% faster race performance (Hoogkamer et al., 2016). There are many reports of linear gross 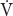
O_2_-velocity relations for treadmill running. The relations have either positive (Pugh, 1970; Léger and Mercier, 1984; Helgerud et al., 2010) or negative 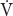
O_2_-intercepts (Joyner, 1991; Daniels and Daniels, 1992; Jones and Doust, 1996), depending on the velocity ranges considered. In the case of a linear gross 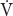
O_2_-velocity relation with a positive 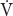
O_2_-intercept, running 1% faster requires *less* than 1% more oxygen. In the case of a linear gross 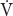
O_2_-velocity relation with a negative 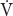
O_2_-intercept, running 1% faster requires *more* than 1% more oxygen. It is therefore critical to base any extrapolation of metabolic savings to running performance on the best available 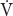
O_2_-velocity relation data.

**FIGURE 1:**
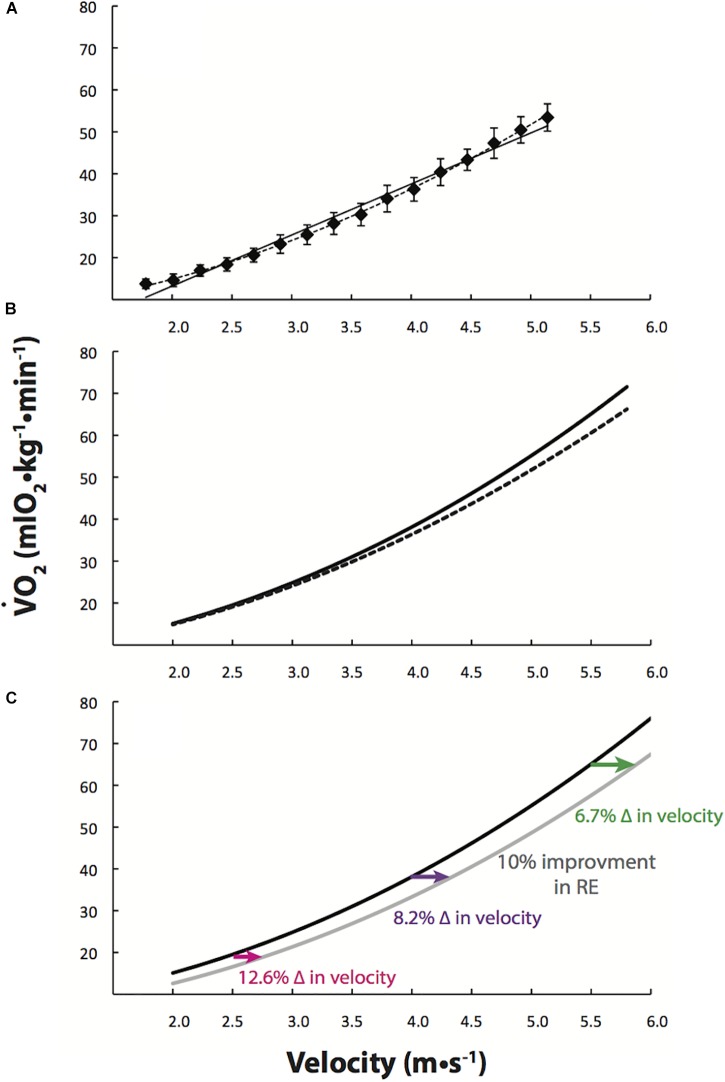
Oxygen uptake (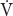
O_2_) increases curvilinearly with running velocity. **(A)** Linear (solid line) and curvilinear (dashed line) regressions to treadmill running data from 10 high-level male runners (<30-min 10 km) over a wide range of velocities (1.78–5.14 m/s) (Batliner et al., 2018). **(B)** Batliner et al. (2018) quadratic equation (dashed line) and the quadratic equation combined with Pugh’s cubic term for overcoming air resistance (solid line), as per Eq. [2]. **(C)** Based on this cubic Eq. [2] (black line), a 10% improvement in running economy (RE; gray line) allows for percent improvements in running velocity which depend on running velocity itself. At slower running velocities (∼<3.0 m/s), 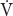
O_2_ increases gradually with increases in running velocity, and, as a result at 2.5 m/s a 10% improvement in RE should facilitate running 12.6% faster. At faster running velocities, 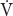
O_2_ increases steeply with running velocity and as a result at 5.5 m/s, a 10% improvement in RE should allow for running only 6.7% faster.

More recent treadmill running studies have indicated that both the gross 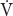
O_2_-velocity relation and the metabolic rate (Watts or kcal/min)-velocity relations are actually better described as inherently curvilinear, especially over wide ranges in velocity (Steudel-Numbers and Wall-Scheffler, 2009; Batliner et al., 2018; Black et al., 2018; Kipp et al., 2018). Figure 1A illustrates both linear and curvilinear regressions to treadmill running data from 10 high-level male runners (<30-min 10 km) for velocities spanning 1.78–5.14 m/s and measured at ∼1600 m altitude (Batliner et al., 2018). The upward curvilinear relation explains why a positive gross 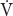
O_2_-intercept is observed when a linear regression line is fitted to slow velocity gross 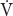
O_2_ data (Bransford and Howley, 1977; Maughan and Leiper, 1983) and a negative intercept when fitted to fast velocity gross 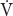
O_2_ data (Conley and Krahenbuhl, 1980; Joyner, 1991; Daniels and Daniels, 1992; Jones and Doust, 1996). A critical implication of a curvilinear gross 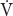
O_2_-velocity relation is that at fast running velocities, a 1% improvement in RE translates to smaller (<1%) improvements in running velocity and thus a less than directly proportional performance benefit. This inherent upward curvilinearity, has not previously been accounted for in models to predict running performance (di Prampero et al., 1986; McLaughlin et al., 2010).

Air resistance is a second important consideration when translating metabolic savings quantified in treadmill studies to overground running performance. Most studies that show a curvilinear 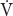
O_2_-velocity relation have actually been conducted on treadmills, with negligible air resistance (Steudel-Numbers and Wall-Scheffler, 2009; Black et al., 2018; Batliner et al., 2018; Kipp et al., 2018). However, as described by Pugh (1970, 1971), the oxygen cost of overcoming air resistance can be expected to increase more than proportionally at faster running velocities, since air drag force is proportional to air (running) velocity squared (du Bois-Reymond, 1925; Hill, 1928) and hence mechanical power (force × velocity) is proportional to velocity cubed. Specifically, Pugh (1971) related the metabolic cost of overcoming air resistance to the mechanical power needed to overcome the air drag forces during running: 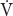
O_2_ (L/min) = 0.00354⋅A_p_⋅*v*^3^ for an athlete of projected frontal area A_p_ (m^2^), running at velocity *v* (m/s), through still air. Throughout this paper, we will use an A_p_ of 0.45 m^2^, for an elite male marathoner (58 kg and 1.71 m) (DuBois and DuBois, 1916; Hoogkamer et al., 2017). Léger and Mercier (1984) added Pugh’s cubic air resistance term to the linear equation they had derived from a regression on data from 10 separate treadmill studies over various moderate velocity ranges. Velocity (*v*) is expressed in m/s for all equations below.

Léger and Mercier, 1984 (including Pugh’s cubic term):

(1) $$\dot{V}O_{2}(ml/kg/min)=0.02724v^{3}+11.39v+2.209$$

In Eq. 2 and Figure 1B (sold line), we added Pugh’s cubic air resistance term to the inherent curvilinear equation from Batliner et al., 2018.

Batliner et al., 2018 + Pugh’s cubic term:

(2) $$\dot{V}O_{2}(ml/kg/min)=0.02724v^{3}+1.5355v^{2}+1.5354v+15.661$$

In Figure 1C we have depicted how this curvilinear 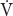
O_2_-velocity relation affects the predicted improvements in running velocity with a consistent hypothetical 10% improvement in RE. The percent velocity enhancement resulting from an improvement in RE depends on the baseline running velocity itself.

## Running Velocity and Potential Improvements in Velocity

Multiple long-term interventions, such as endurance, interval, resistance, and plyometric training, have been shown to improve RE (for review: Saunders et al., 2004; Barnes and Kilding, 2015). Other factors such as the racecourse elevation profile (e.g., downhill) (Minetti et al., 2002), favorable meteorological conditions and innovations in footwear can also improve RE (Hoogkamer et al., 2017). Recently, we showed that a prototype of the Nike Vaporfly 4%, a shoe with exceptionally compliant and resilient midsole in which a stiff carbon-fiber plate is embedded improved RE by an average of 4%, compared to two well-established racing shoes (Hoogkamer et al., 2018a). The mechanisms behind the energy savings have been detailed in Hoogkamer et al. (2019). How much faster could an athlete wearing these shoes run, assuming their response is equal the average response of our group; i.e., a consistent improvement in RE of 4%?

**FIGURE 2:**
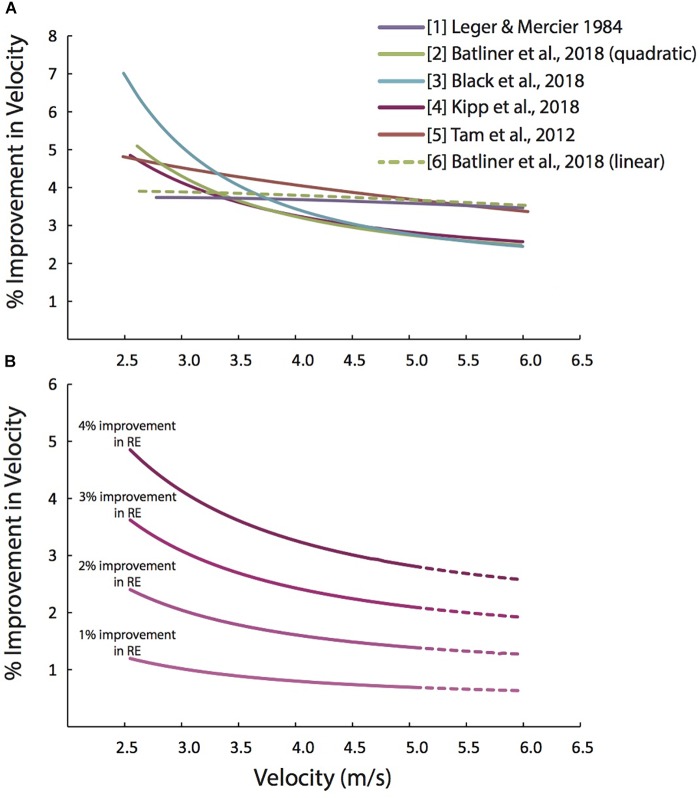
Predicted percent improvements in running velocity depend on the baseline running velocity. **(A)** Predicted percent improvements in running velocity vs. running velocity, based on a 4% improvement in RE, using several equations from the recent scientific literature. The solid green is based on a quadratic fit through Batliner et al. (2018) data with Pugh’s cubic air resistance term. The green dashed line is based on a linear fit through Batliner et al. (2018) data combined with Pugh’s cubic air resistance term. The difference between the two green lines highlights the importance of the inherent curvilinearity of the 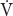
O_2_-velocity relation which substantially alters the magnitude of percent improvement in velocity. **(B)** Predicted percent improvements in running velocity vs. running velocity, based on 1 to 4% improvements in RE, using Eq. [2], which combines the quadratic equation from Batliner et al. (2018) with Pugh’s cubic air resistance term. Beyond the velocity range of Batliner et al. (2018) (>5.14 m/s) prediction lines are dashed.

We quantified the possible improvements in running velocity using Eqs 1 and 2, by Léger and Mercier (1984) and Batliner et al. (2018), treadmill data from Black et al. (2018) and Kipp et al. (2018), and overground running data from Tam et al. (2012). Figure 2A shows how the improvements in running velocity that are possible with a 4% improvement in RE depend on running velocity for each of these studies.

We fit a quadratic equation through the 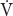
O_2_ data of Black et al. (2018) who studied 14 male and 10 female athletes running at 10 different velocities from 2.22 to 4.72 m/s at sea level. Then, we added Pugh’s cubic air resistance term (Eq. 3).

Black et al., 2018 + Pugh’s cubic term:

(3) $$\dot{V}O_{2}(ml/kg/min)=0.02724v^{3}+1.9128v^{2}+3.2483v+25.806$$

Similarly, we fit a quadratic equation through Kipp et al.’s (2018) data measured at ∼1600 m altitude for 10 high-level male athletes at six running velocities ranging from 2.22 to 5.00 m/s and added Pugh’s cubic air resistance term (Eq. 4).

Kipp et al., 2018 + Pugh’s cubic term:

(4) $$\dot{V}O_{2}(ml/kg/min)=0.02724v^{3}+1.7321v^{2}+0.538v+18.91$$

Uniquely, Tam et al. (2012) measured 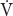
O_2_ in 10 elite male Kenyan athletes (<2:09-h marathon) running overground on a clay track at ∼2,000 m altitude at four running velocities ranging from 3.33 to 5.00 m/s. They constrained their regression to have a linear and a cubic term, without a square term, similar to Léger and Mercier (1984). Tam et al. (2012) expressed their metabolic data in net energy cost of transport (J/kg/km) (gross – upright resting) and then fit a line through the data plotted against the square of velocity. We repeated this analysis for the data expressed in ml O_2_/kg/km, converted this to rate of oxygen uptake in ml O_2_/kg/min and then added the reported upright resting rate of oxygen uptake, to get gross 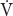
O_2_ values at each velocity (Eq. 5).

Tam et al., 2012:

(5) $$\dot{V}O_{2}(ml/kg/min)=0.0537v^{3}+9.8158v+5.7$$

The equations with a square term (Eqs. 2–4) all follow a similar trend and concur closely for running velocities faster than 4 m/s (Figure 2A). While the cubic term in Eq. 1 is identical to that in Eqs. 2–4, Eq. 1 predicts fairly consistent velocity improvements over the presented velocity range (2.5–6.0 m/s), as opposed to the increasingly smaller percent velocity improvements predicted with Eqs. 2–4. This indicates that the square term (which represents the inherent curvilinearity of the 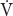
O_2_-velocity relation) substantially alters the relation between baseline running velocity and the possible improvements in running velocity. This is also demonstrated by the dashed line, which is based on a linear fit through Batliner et al.𡀙s (2018) data with Pugh’s cubic air resistance term added.

Linear fit of Batliner et al., 2018 + Pugh’s cubic term:

(6) $$\dot{V}O_{2}(ml/kg/min)=0.02724v^{3}+12.2v+1.11$$

Interestingly, this line closely resembles the running velocity improvements predicted using Leger and Mercier’s (1984) and Tam et al.𡀙s (2012) equations, which do not have a square term. In short, ignoring the inherent curvilinearity of the 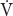
O_2_-velocity relation results in over-prediction of the percent improvements in velocity at the faster velocities.

It is important to realize that Eqs. 1–4 are used to predict changes in performance at sea level. If one wants to apply these equations to predict changes in performance at other altitudes, Pugh’s cubic air resistance term should be adjusted for the difference in air density. While second order polynomials are fit through treadmill 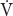
O_2_ data collected at altitude (∼1600 m for Batliner et al., 2018 and Kipp et al., 2018), we believe that the effect of air density on the relation between 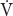
O_2_ and treadmill running speed is small, since externally it would only affect the cost of moving the extremities through the air relative to the torso.

Eqs. 2–4 take into account the inherent curvilinearity of the 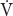
O_2_-velocity relation, but their coefficients differ slightly. This is likely due to differences in the subject populations tested and the experimental setups. One of the major determinants of the equation coefficients is the velocity range over which the data were collected. Narrower velocity ranges result in less pronounced curvilinearity of the 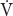
O_2_-velocity relation. A narrower velocity range is what has led many previous studies to describe the 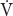
O_2_-velocity relation as linear (Menier and Pugh, 1968; Daniels and Daniels, 1992; Helgerud et al., 2010). Here, we have utilized the Batliner et al., 2018 equation (Eq. 2) because it is derived from the widest running velocity range. Interestingly, even though it was collected over the widest range of velocity, it has the most conservative inherent curvilinearity term (as seen in the square term of the equation).

## Implications for Running Performance

Figure 2B depicts the relation between the baseline running velocity and the percent increases in running velocity possible for different percent improvements in RE, based on Eq. 2. With an improvement in RE of 1% (due to training, footwear, nutrition, tailwind, etc.) a recreational athlete who could typically run at 2.60 m/s (4:30:00 marathon) would be predicted to run their race 1.17% faster, finishing in ∼4:26:53, a 3 min and 7 s improvement. Alternatively, with the same 1% improvement in RE, an elite marathoner running at 5.72 m/s (2:03:00 marathon), would be able to run only 0.65% faster, finishing in 2:02:13, only a 47 s improvement. A similar trend is apparent for all improvements in RE (Figure 2B). Generally, for velocities slower than ∼3 m/s, the percent improvement in velocity are expected to be slightly greater than the percent improvement in RE. For velocities faster than ∼3 m/s, percent improvements in velocity are expected to be less than the percent improvements in RE. At velocities faster than ∼5.5 m/s (∼2:08 marathon pace), percent improvements in velocity are expected to be less than 2/3rds of the percent improvements in RE.

We used this same approach to go back to our 2016 study (Hoogkamer et al., 2016), where we demonstrated that lab-measured changes in RE translate to similar changes in distance running performance, assessed by 3 km time trials. The metabolic data indicated that adding 100 g mass to each shoe worsened RE on average by 1.11%, while it slowed 3 km time trial performance by 0.78%. The discrepancy in those percent changes can now be explained by the inherent curvilinearity of the 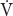
O_2_-velocity relation and the additional curvilinear cost of overcoming air resistance. Eq. 2 predicts that a 1.11% worsening in RE at a running velocity of 4.79 m/s (i.e., the average running velocity during the 3 km time trials) would result in a 0.78% slower time, exactly matching the experimentally observed average slowing of the time trial performances.

Calculating predicted improvements in running velocity based on baseline running velocity and percent improvements in RE based on Eq. 2, requires the non-trivial solving of a third-order polynomial for running velocity (*v*). To allow readers to calculate their own comparisons/predictions, we provide a spreadsheet that solves the cubic equation (see Supplementary Material). The spreadsheet predicts marathon, half-marathon, and 10 km performances based on only four inputs: height, weight, percent improvement in RE and baseline performance. When using this calculator, it is important to realize that it provides a general prediction that does not take into account individual variability in the 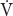
O_2_-velocity relation. Furthermore, percent improvements in RE due to footwear innovations (Hoogkamer et al., 2018a) or long-term training interventions (Saunders et al., 2004; Barnes and Kilding, 2015) also differ between individuals. Finally, Pugh’s cubic air resistance term is dependent on a runner’s projected frontal area, which can be estimated based on the runner’s height and body mass. In this paper, we have assumed those to be 1.71 m and 58 kg, respectively. In the Supplementary Spreadsheet, these numbers can be adjusted at will.

At the previous world record marathon pace of 5.72 m/s, a 4% improvement in RE translates to a 2.64% faster running velocity, allowing a marathon time of 1:59:47. Yet, with the introduction of a 4% more economical running shoe, the marathon world record has only been broken by 1.03%. It is important to note that Dennis Kimetto, the previous holder of the world record has not competed in the newly developed shoe. The fastest marathon by Eliud Kipchoge (current marathon world record holder) prior to adopting the shoes with an average of 4% RE enhancement was 2:04:00 at Berlin in 2015. According to our calculations, starting with a 2:04 baseline, a 3% improvement in RE translates to a 1.97% faster velocity or 2:01:36, almost exactly equal to the recently set world record. It is unknown how much of a RE enhancement Kipchoge experiences in the new shoes.

## Possible Confounding Factors

The major assumption in our approach to predict improvements in running performance based on improvements in RE is that all other performance related factors remain the same. This might not always be the case. For example, when RE is improved through drafting behind other competitors or pacemakers, the reduced air flow over the skin might negatively affect the runner’s thermoregulation (less heat convection/evaporation), which could impair running performance and, at least partly, counter the gains in RE (Hoogkamer et al., 2018b). Although small body size provides thermoregulatory advantages (via a greater surface area to volume ratio) (Joyner et al., 2011), the aerodynamic drag force per kg body mass is greater for smaller individuals.

Similarly, when RE is improved by running an overall downhill course, it can be expected that the repeated eccentric loading will result in additional muscle damage (Hikida et al., 1983), which will negatively affect running performance. Muscle damage is likely to occur in elite marathon runners due to the distance and fast speeds, but it is not well understood how RE changes with muscle damage or fatigue. Indeed, there are several reports of worsening RE during the marathon and ultra-marathon distance (Petersen et al., 2007; Vernillo et al., 2017), which might be related to muscle damage, fatigue (Millet et al., 2011) or substrate utilization shifts (Vernillo et al., 2017). However, as long as those RE changes during the marathon are consistent and do not change the curvilinearity of the 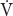
O_2_-velocity relation, deterioration in RE during a race should not affect our predictions. Theoretically, running faster *per se*, independent of the source of the improvement in RE, might result in more muscle damage during a race, which would impair running performance. However, more cushioned running shoes can be expected to reduce muscle damage. It may also be that the extensive training of elite marathoners mitigates the muscle damage common in slower marathoners. Further, some data suggest that RE differences between shoes might be affected by fatigue (Vercruyssen et al., 2016).

A potential limitation of our approach is that we do not have direct measurements of the relation between running velocity and metabolic energy cost (i.e., W/kg or kcal/min) at elite marathon pace. If this relation is steeper beyond the tested velocity range, percent improvements in performance will be even smaller. Distance runners in shorter races (e.g., half-marathons and 10 km) compete at velocities above their lactate threshold, where it is not possible to measure RE due to contributions from non-oxidative sources. It is not completely understood how the total metabolic demands (oxidative and non-oxidative) change at these high intensities.

Unlike elite runners, slower runners should have a greater percent improvement from technological advancements in footwear. As shown in Figure 1C, at slower speeds, there is a greater improvement in velocity for a given improvement in RE. Thus, it is likely that improvements in RE from footwear will produce a wave of recreational runners setting personal records (Quealy and Katz, 2018).

## Future Perspectives

Our analysis here focused solely on the oxygen cost of running. Expressing RE in units of rates of energy utilization (W/kg or kcals/min/kg) accounts for differences in substrate utilization and, therefore, in the amount of energy liberated per liter oxygen. To be most relevant to elite marathoners, future investigations should quantify how the energy cost of running changes during overground running at world-class marathon velocities on pavement surfaces at sea level.

## Author Contributions

SK and WH were responsible for conception of the review. SK drafted the manuscript. RK and WH revised it. RK conceived of the calculator in the Supplementary Material while SK and WH developed it. SK, RK, and WH approved the final version of the manuscript. All authors agreed to be accountable for all aspects of the work.

## Conflict of Interest Statement

RK is a paid consultant to Nike, Inc. The remaining authors declare that the research was conducted in the absence of any commercial or financial relationships that could be construed as a potential conflict of interest.

## Footnotes

1 We and others prefer to express RE in units of energy utilization (W/kg or kcals/min/kg) (Fletcher et al., 2009; Shaw et al., 2014; Beck et al., 2018; Kipp et al., 2018) to account for differences in substrate utilization and therefore, in the amount of energy liberated per liter of oxygen uptake. Here, we needed to incorporate several classic studies that only reported oxygen uptake rates and thus we express RE in units of oxygen uptake rate.
